# Asymmetric and Spiraled Genitalia Coevolve with Unique Lateralized Mating Behavior

**DOI:** 10.1038/s41598-020-60287-w

**Published:** 2020-02-24

**Authors:** Dara N. Orbach, Patricia L. R. Brennan, Brandon P. Hedrick, William Keener, Marc A. Webber, Sarah L. Mesnick

**Affiliations:** 1 Texas A&M University- Corpus Christi, Department of Life Sciences, 6300 Ocean Dr., Corpus Christi, Texas, 78412 USA; 2 Mount Holyoke College, Department of Biological Sciences, 50 College Street, South Hadley, Massachusetts 01075 USA; 3 Louisiana State University Health Sciences Center, Department of Cell Biology and Anatomy, 1901 Perdido Street, New Orleans, LA 70112 USA; 4 University of Oxford, Department of Earth Sciences, South Parks Road, Oxford, OX1 3AN UK; 5 The Marine Mammal Center, 2000 Bunker Road, Sausalito, California 94965 USA; 6 Marine Mammal and Turtle Division, Southwest Fisheries Science Center, National Marine Fisheries Service, National Oceanic and Atmospheric Administration, 8901 La Jolla Shores Drive, La Jolla, California 92037 USA

**Keywords:** Coevolution, Sexual selection

## Abstract

Asymmetric genitalia and lateralized mating behaviors occur in several taxa, yet whether asymmetric morphology in one sex correlates or coevolves with lateralized mating behavior in the other sex remains largely unexplored. While lateralized mating behaviors are taxonomically widespread, among mammals they are only known in the harbor porpoise (*Phocoena phocoena*). Males attempt copulation by approaching a female exclusively on her left side. To understand if this unusual lateralized behavior may have coevolved with genital morphology, we quantified the shape of female and male harbor porpoise reproductive tracts using 2D geometric morphometrics and 3D models of the vaginal lumen and inflated distal penis. We found that the vaginas varied individually in shape and that the vaginas demonstrated both significant directional and fluctuating asymmetry. This asymmetry resulted from complex 3D spirals and vaginal folds with deep recesses, which may curtail the depth or direction of penile penetration and/or semen movement. The asymmetric shapes of the vaginal lumen and penis tip were both left-canted with similar angular bends that mirrored one another and correspond with the left lateral mating approach. We suggest that the reproductive anatomy of both sexes and their lateral mating behavior coevolved.

## Introduction

Left- or right-bias in morphology and behavior in otherwise bilaterally symmetrical animals manifests in diverse biological phenomena such as mating, foraging, predation, predator defense, and communication^1,2^. Asymmetries in genital morphology are known in several animal taxa^3–5^, and although lateralization in courtship behavior is found across animal taxa^6^, it was unknown in mammals until recently^7^. While some morphological and behavioral asymmetries related to mating have been identified, the relationship between genital asymmetry and lateralized mating behaviors, as well as their evolutionary significance, has rarely been examined. Questions remain about how asymmetry in one sex influences the behavior or morphology of the other sex and whether asymmetries arise from adaptive (directional evolution) or non-adaptive (genetic drift) mechanisms.

Instances in which one or both sexes have asymmetries in both mating behavior and genitalia may be more common than is currently recognized. For example, lateralized mating behavior occurs in male poeciliid fish, which angle their gonodopodium (intromittent organ) to either the left or right side and are restricted to mating with females that have a genital opening on the same side^8–10^. Similarly, male tree swallows tend to copulate from the left, perhaps because the female’s one active oviduct is on the left side^11^. Male earwigs with paired penises preferentially use their right penis during copulation, which may be driven by female genital morphology^12^. Male and female waterfowl have asymmetric genitalia that spiral in opposite directions and females have evolved behavioral strategies to influence control over insemination^13–15^. However, mating behaviors are not lateralized in either waterfowl sex.

Female cetartiodactyls (cetaceans and even-toed ungulates) are unusual in possessing vaginal folds, protrusions of the vaginal wall into the vaginal lumen, which may function in sexual selection, among other hypotheses^16,17^. Additionally, among mammals, asymmetric penises are most common in cetartiodactyls^4^, which have a fibroelastic penis that maintains a turgid state and is resistant to bending^18^. Both male and female genital morphology in cetaceans is particularly complex. The penis tip of cetaceans is pliable and may be under voluntary control^19,20^. Female harbor porpoises (*Phocoena phocoena*) have one of the most complex vaginal morphologies and the largest number of vaginal folds described in any cetacean species^16^. Male harbor porpoises have a large blunt knob at the terminal end of the relatively long penis shaft (among the proportionally longest in cetaceans), which connects to a thin distal tip that bends away from the midline^19^. During copulation, the blunt knob may be obstructed from penetrating the cranial vagina by the largest vaginal fold, as suggested by *ex vivo* testing^21^. The thin distal penis tip has been hypothesized to enable passage between the remaining cranial vaginal folds to deposit sperm close to the cervix^21^. The vaginal folds may also dampen biomechanical forces to surrounding tissues during copulation^22^. The close biomechanical interaction and fit between male and female genitalia in general during copulation suggests that reproductive morphology may coevolve^15^.

Harbor porpoises are currently the only known mammal with lateralized mating behavior^7^. Although asymmetries in marine mammal behavior tend to be right-biased (e.g., feeding^23^), recent documentation of 142 copulation attempts by male harbor porpoises in San Francisco Bay showed they were all performed on the female’s left side^7^. Copulatory attempts consisted of rapid (1–4 seconds), high-energy, precision-timed approaches, during which the males often breached out of the water with their penis fully extended and attempted to drive their penis into the female’s vaginal opening^7^. The complexity of their genital morphology and their lateralized mating behavior make the harbor porpoise an exemplary taxon to explore the potential relationship between reproductive morphology and mating behavior. To characterize and quantify genital shape and assess the influence of asymmetry on overall genital shape, we used two-dimensional geometric morphometrics (2DGM) and three-dimensional models of male and female harbor porpoise genitalia. Asymmetry has been previously reported in female genitalia in a wide range of taxa, but can be inconspicuous when present^3–5,13,24^. Therefore, we examined the pattern of asymmetry in vaginal folds in both 2D and 3D by generating models of the vaginal lumen, in addition to assessing gross morphology. This is the first study to address the question of whether lateralized mating behaviors affect genital morphology in mammals, and allows for a better understanding of the drivers of sexual patterns in both cetaceans and the wide variety of animals that have asymmetric genitalia with potentially previously unrecognized lateralized mating behaviors.

## Methods

Female (n = 10) and male (n = 3) reproductive tracts were obtained from fresh or moderately decomposed deceased sexually mature harbor porpoises that died of natural causes or fisheries interactions in the San Francisco Bay Area. Specimens were frozen and shipped from The Marine Mammal Center to necropsy facilities at either Mount Holyoke College or the National Oceanic and Atmospheric Administration’s Southwest Fisheries Science Center. Specimens were obtained under a National Marine Fisheries Service salvage permit to D.N.O. or S.L.M. Female reproductive tracts consisted of the external genital opening through to the uterine horns and ovaries^25^. Male reproductive tracts included the entire penis tip and shaft to the pelvic bones. Sperm was found in the testes of all male specimens, confirming their sexual maturity at the time of death. Specimen information (i.e., body length, weight, and female reproductive state) was provided by The Marine Mammal Center (Table 1).

**Table 1:** Sex, reproductive state, and body sizes of the post-mortem harbor porpoises.

ID in Fig. 3	Specimen ID	Sex	Reproductive State	Body Length (cm)	Body Weight (kg)
1	C-434	Female	Lactating	174	68
2	C-358	Female	Pregnant	158	Not Measured
3	C-383	Female	Pregnant	169	Not Measured
4	C-392	Female	Lactating	163	Not Measured
5	C-399	Female	Resting	153.5	67
6	C-403	Female	Pregnant	146	51
7	C-405	Female	Resting	164	72
8	C-415	Female	Pregnant	164	55
9	C-431	Female	Resting	172	53
N/A	C-441^*^	Female	Resting	143	41
N/A	C-485	Male	N/A	153	59
N/A	C-494	Male	N/A	90	61.5
N/A	C-496	Male	N/A	147	61.5

^*^Specimen positioned in ventral recumbency, unlike the other female specimens.

Nine female reproductive tracts were dissected in dorsal recumbency (the specimen lying on its dorsal side with the ventral surface exposed). The reproductive tract is oriented horizontally (cranial-caudal) in the female’s body. As vaginal folds are most prominent on the dorsal side of the vagina^25^, incisions were made on the ventral side to visualize the shape of internal structures^21^. A single incision was made down the ventral midline of each reproductive tract, from the internal bifurcation of the uterine horn through the clitoris^25^. Measurements of the thickness of the largest vaginal fold were collected with digital calipers (Mitutoyo 500-171 Digital Calipers). Photographs were taken of the open reproductive tracts in a standardized bird’s-eye view using digital cameras with a minimum resolution of 10.1 megapixels. The focal plane of the camera was positioned parallel to the midline of the reproductive tract, with the long axis of the vaginal canal oriented such that the vaginal opening was at the bottom of the image. Scales were positioned in both transverse and coronal (frontal) planes.

**Figure 1:**
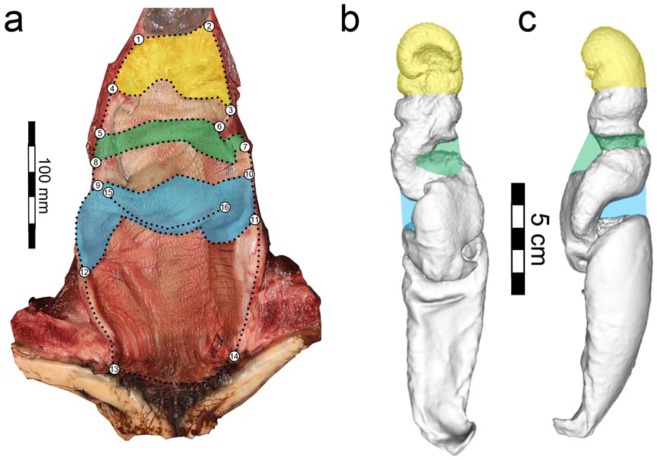
(**a**) Landmarks (numbered) and semi-landmark curves (black dots) of a representative adult harbor porpoise vagina with her ventral side exposed (dorsal recumbency). Semi-landmark curves were placed to capture the cervix (yellow), first large cranial fold (green), and major caudal fold (blue). Scale = 100 mm. 3D model of the silicon mold of the vaginal lumen depicted on the (**b**) ventral side, and (**c**) left lateral side (ID: C-434). Scale = 5 cm.

Two-dimensional geometric morphometrics were employed to quantify variation in the shape of the cervix, first large cranial vaginal fold, large main vaginal fold, and vaginal entrance. The cranial and caudal bounds of each region were outlined using both landmarks (n = 16) and semi-landmark curves (n = 20 curves, 240 semi-landmarks) in TPSDig2 (Fig. 1a)^26^. All landmarks were positioned by one author (D.N.O.) to eliminate inter-observer error^5,17,27,28^. As positioning of landmarks in soft tissue can be challenging, they were applied to each image three independent times to quantify intra-observer measurement error. Landmark configurations were imported into R^29^, where a Generalized Procrustes Analysis (GPA) was performed to rescale, translate, and rotate all landmark configurations into the same shape space^30^ using the *geomorph* package^31^. Semi-landmark curves were slid to minimize the bending energy between semi-landmarks^32^. Landmark configurations were then subjected to principal component analysis (PCA) to visualize the major axes of shape change among the specimens and to ensure that measurement error triplicates plotted more closely to one another than to other individuals. Although total length did not vary substantially within the harbor porpoises (146–174 cm), the effect of size on the shape data was tested using the Regression Score metric with log-transformed total length as a proxy for the size of each individual^33,34^.

To quantify 2D asymmetry, a multi-factor ANOVA was used with individuals (symmetric variation), sides (directional asymmetry), and the interaction between individuals and sides (fluctuating asymmetry) as factors^35,36^. Directional asymmetry captures asymmetry where one side is consistently shaped differently from the other (e.g., fiddler crab claws^37^). Fluctuating asymmetry captures random right-left perturbations from symmetry that are a result of developmental or environmental factors^36^. The proportion of total variation explained by each individual factor was calculated using the η^2^ effect size metric^38^.

**Figure 2:**
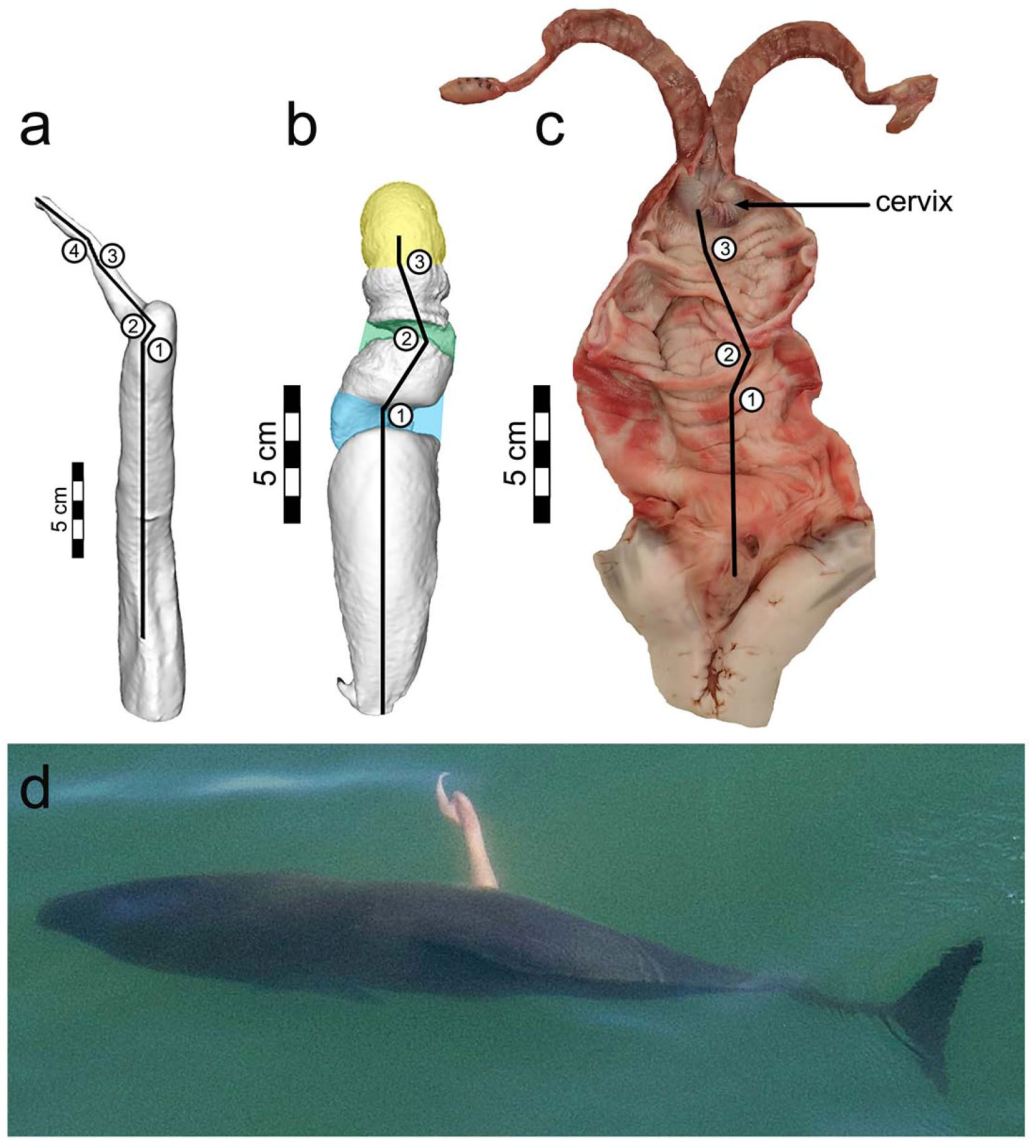
Shape of harbor porpoise penis and pathway through the vaginal lumen. (**a**) Dorsal view of 3D model of inflated portion of the penis angled in a coronal plane with the tip facing cranially (ID: C-485). Straight black lines with numbers demarcate angular changes calculated in Table 2. (**b**) 3D model of the silicon mold of the vaginal lumen depicted on the dorsal side. Straight black lines and numbers correspond to major qualitative changes in orientation of the vaginas (ID: C-434). (**c**) Female reproductive tract with dorsal side exposed (ventral recumbency) with an incision made down the dorsal midline (ID: C-441). Straight black lines show the pathway from the vaginal opening to the cervix around the complex vaginal folds. Numbers represent the major qualitative changes in orientation of the vagina. (**d**) Erect penis of free-swimming harbor porpoise immediately after a copulation attempt depicting angles of the penis.

To capture 3D asymmetry of the vaginal canal, endocasts were made of the lumens prior to dissections. One endocast was made from a female collected from San Francisco Bay and four endocasts were made from adult females obtained from other populations along the Pacific coast of the United States. Female reproductive tracts were suspended and filled with Mold Star® 16 FAST silicone. The endocasts were carefully removed to prevent damage to the molds or tissue and to identify the corresponding ventral and dorsal sides of the female. 3D models of the endocasts were then generated with photogrammetry. The complete circumference of each endocast was photographed using a Canon EOS Rebel T5i camera with a 100 mm lens and was illuminated with four LED lights. Three-dimensional models were reconstructed in 3DF Zephyr lite (3Dflow SRL, Verona, Italy) and scaled and bisected in sagittal and lateral planes using Autodesk^®^ ReCap Photo (v.22.0. San Rafael, CA: Autodesk; 2018). The different planes were qualitatively compared to determine the degree of symmetry. The pathway from the vaginal opening to the cervix was digitally superimposed onto the image (Fig. 2b).

The penis tips and shafts of three male harbor porpoises were filled to distention with melted Vaseline (Unilever) to recreate the likely shape attained during erection (Table 1). Vaseline was injected into the corpora cavernosa and corpora spongiosa tissues with an 18-gauge needle connected to a syringe until turgidity and resistance to further distension was achieved (e.g.^21,39^). The artificially inflated penises were engorged to similar dimensions as observed in several photographs of free-swimming harbor porpoises with erections (Fig. 2d)^7^. 3D models were made using photogrammetry with the same methods described above for the vaginal endocasts. The digital penis models were angled in a consistent coronal plane with the tip facing cranially using Autodesk^®^ ReCap Photo. A black line was drawn down the dorsal midline of each 3D penis model (from the attachment of the retractor muscle to the distal tip) to calculate the angles of each bend in the penis tip relative to the midline which was set at 0 degrees (Fig. 2a). Major changes in penis angles were calculated relative to the preceding angles using the angle tool in ImageJ (v.1.52k^40^).

One female reproductive tract was dissected in a ventral recumbency (the specimen lying on its ventral side with the dorsal surface exposed) with an incision made down the dorsal midline (Table 1; ID C-441). This orientation reflects the natural position of females in the wild (ventral side down) during male left-sided sexual approaches^7^. The pathway from the vaginal opening to the cervix around the complex vaginal folds was digitally superimposed onto an image of this dissected reproductive tract (Fig. 2c). We then qualitatively compared the superimposed pathway through the vaginal lumen with the shape of the inflated penises (Fig. 2a).

### Significance statement

We explored the coevolution of genital morphology and mating behavior asymmetry between male and female harbor porpoises, the only mammal known to demonstrate exclusive lateralized mating behavior. By comparing 2D and 3D shapes of excised male and female reproductive tracts, we found that genital asymmetry is likely functional. Male and female genital shapes are congruent in angular bends and a left-sided sexual approach by males is obligatory to overcome physical obstructions inside the female reproductive tract. Our results suggest male copulatory behavior and morphology conform with female morphological modifications to achieve fertilization in a system which may undergo an evolutionary arms race.

## Results

**Figure 3:**
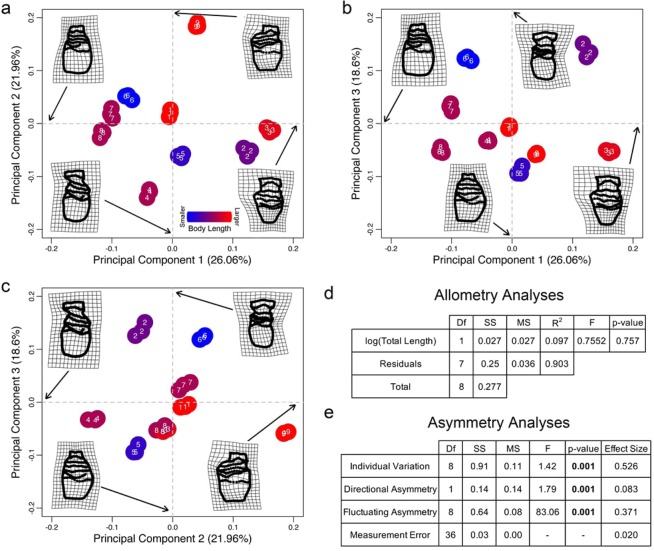
Principal component analysis of nine vaginas with three replicates each showing the variability of vaginal shape within the morphospace (visualized using thin-plate spline grids) comparing (**a**) PC1 and PC2, (**b**) PC1 and PC3, and (**c**) PC2 and PC3. Specimens are colored based on body length (hotter colors equal longer porpoises) showing a limited relationship between specimen body length and vagina shape. (**d**) Procrustes ANOVA of total body length and shape. (**e**) Asymmetry analyses showing p-values and effect sizes for the symmetric component of variation (individual variation), directional asymmetry, fluctuating asymmetry, and measurement error.

We found substantial variation in vaginal shape in 2D using PCA. Principal component (PC) 1 (26.06% of total shape variation) was driven by the relative size of the lower vagina (caudal to the largest vaginal fold; Fig. 3a), PC2 (21.96% of total shape variation) was driven by a left- or right-biased curvature of the entire reproductive tract (Fig. 3a), and PC3 (18.6% of total shape variation) was driven by the relative widths of the cervix and vagina (Fig. 3b,c). Vaginal shape was not significantly correlated with total body length (p = 0.757; Fig. 3d). Based on ANOVA effect sizes, individual variation accounted for 52.6% of total shape variation, directional asymmetry for 8.3% of total shape variation, and fluctuating asymmetry for 37.1% of total shape variation. All three of these factors were significant (p < 0.001; Fig. 3e). Measurement error, by contrast, only comprised 2% of the total shape variation, demonstrating that the landmarking process did not generate a substantial error component.

The internal vaginal lumen is highly asymmetric due to both complex 3D spirals and vaginal folds with deep recesses (Fig. 1b,c). The largest vaginal fold had a mean thickness of 19.6 mm (S.D. = 4.7 mm, n = 8). The pathway through the vaginal lumen to the cervix had an overall bend and spiraled to the left in all five endocasts when positioned in ventral recumbency, as found during mating under natural conditions (Fig. 2b). This bend, and two additional moderate bends in the vaginal lumen, are generated by the vaginal folds. When viewed dorsally, the tips of all three inflated penises originate on the left side of the blunt knob and cant to the left in a series of four consecutive bends (Table 2; Fig. 2a). Specifically, relative to the midline of the long axis of the shaft, the penis angles marginally to the right slightly proximal to the blunt knob, then bends ~90° to the left and downwards toward the shaft, followed by subtler bends to the right then left near the distal tip. These bends parallel the shape of the internal vaginal lumen (Figs. 1c and 2b) and accurately reflect the penis shape of free-swimming harbor porpoises in San Francisco Bay (Fig. 2d) when males attempt copulation. A video of a copulatory attempt shows the body orientation of males relative to females (Supplemental Video 1).

**Table 2:** Angles of the inflated penis tips of three harbor porpoises.

Specimen ID	Angle 1	Angle 2	Angle 3	Angle 4
C-485	35° to right	76° to left	26° to right	30° to left
C-494	40° to right	75° to left	27° to right	45° to left
C-496	40° to right	97° to left	61^o^ to right	37^o^ to left

The first angle is relative to the long axis of the penis shaft such that 0° angles parallel to shaft in the cranial direction and the subsequent angles are relative to the preceeding angle. The four angles show marked directional changes in the penis tip (Fig. 3a). Prior to measurement, penises were angled in a coronal plane with the tip facing cranially.

## Discussion

We provide evidence that genital asymmetry has coevolved with lateralized mating behavior in harbor porpoises, the first example amongst mammals. The vaginal lumen of harbor porpoises is significantly directionally asymmetric, and its shape corresponds to the leftward cant of the erect penis tip. The left-sided mating behavior of male harbor porpoises appears to have coevolved with genital shape variation in both sexes so that the penis can circumvent large and protruding vaginal folds^21^. The close genital correspondence could be the result of several potential coevolutionary mechanisms, including sexual conflict, female choice, and “lock-and-key”. As hybridization is common among cetaceans^41^, it is unlikely a “lock-and-key” mechanism, and the close correspondence likely arises as a result of an intersexual evolutionary arms race in harbor porpoises. In the San Francisco Bay population, males initiate all mating events, attempt copulations throughout the year, do not engage in pre-copulatory courtship, and approach females in all reproductive states^7^. Females may respond to the constant pursuit of males by allowing copulations that reduce costs of evasion and harassment. However, they have also evolved sophisticated mechanisms that may control paternity including asymmetric genitalia, complex vaginal folds, changes in body positioning during copulation that may function to misalign the optimal angle of penetration and prevent the ejaculate from reaching the cervix, and through resistant behaviors (e.g., dives^42^). Both sexes appear to exhibit both behavioral and anatomical adaptations and counter-adaptations that may control paternity, although it is possible that an evolutionary arms race has not reached an equilibrium end point (e.g.^43^). It is clear that penile penetration into the most cranial regions of the vagina requires a specific angle of entry to bypass the vaginal folds. By angling their bodies slightly when males approach, females could potentially prevent the tip of the penis from penetrating past their vaginal folds^21^. Males seem to have evolved complex penile morphology with numerous angles that match the internal shape of the vagina and may increase their chances of successful copulation. The large relative testes size of harbor porpoises compared to other cetaceans suggests a substantial investment in post-copulatory sperm competition^44,45^ and there is likely strong selection on females to evolve mechanisms to control paternity after insemination by multiple males.

Although all the female reproductive tracts examined were asymmetric and complex, we found that vaginal shape strongly varied among individuals. Female genitalia were previously considered to be less variable than male genitalia^46^, yet emerging patterns suggest a high level of intra- and inter-specific variation in females, which highlights the need to continue to explore female genital morphological variation across taxa^47^. Reproductive state and history could have contributed to this variation, but our sample size was too small to examine this possibility quantitatively. For example, a study on reproductive tract lengths in common bottlenose dolphins (*Tursiops truncatus*) found that pregnant females had longer vaginal lengths than lactating or resting adult females, although the sample size was also too small for statistical analysis^25^. We qualitatively note that pregnant harbor porpoises generally had more symmetrical cervices, potentially reflecting stretching, while resting females had more asymmetrical cervices. Recent assessment of soft vaginal structures in other taxa using 2DGM have similarly found that vaginal shape is highly variable and influenced by factors such as ontogeny and reproductive state (beetles^48^; snakes^49^; cetaceans^17^; sharks^5^). However, much of the documented variation remains unexplained.

More than 8% of total shape variation in the harbor porpoise vagina was explained by directional asymmetry. As directional asymmetry is often functional^50,51^ and all vaginal endocasts canted to the left, the data support a left-sided constraint on males to approach females only from one direction and body orientation. We found that fluctuating asymmetry was significant and explained 37% of total variation. This high degree of fluctuating asymmetry in soft tissue structures is common (e.g., dogfish shark vagina^5^), and may result from less canalization in comparison with rigid hard tissues^52,53^. While genital laterality is to the left, other morphological asymmetries known in harbor porpoises are to the right (e.g., flipper size^54^; nasal complex asymmetries^55,56^), and right-dominated asymmetries in non-mating behavior occur in other marine mammals^23^. This makes it likely that the genital asymmetry is adaptive under the influence of selection, and does not result from pleiotropy in harbor porpoises, which could favor right-sided development concurrently with other right-sided morphological asymmetries.

We have demonstrated the development and coevolution of lateralized sexual behavior and genital morphology in harbor porpoises, which appears to reflect an intersexual evolutionary arms race. It is not yet possible to determine whether the lateralized behavior evolved in response to morphological evolution or vice versa, or how it became fixed in the species. Substantial future work on additional species is necessary to establish the commonality of correlations between lateralized behavior and morphology among vertebrates.

## Supplementary information

Supplemental video 1.

## References

[1] Hori M (1993). Frequency-dependent natural selection in the handedness of scale-eating cichlid fish. Science.

[2] Bisazza A, Rogers LJ, Vallortigara G (1998). The origins of cerebral asymmetry: a review of evidence of behavioural and brain lateralization in fishes, reptiles and amphibians. Neurosci. Biobehav. Rev..

[3] Schilthuizen M (2007). The evolution of chirally dimorphic insect genitalia. Tijdschr. Ent..

[4] Schilthuizen M (2013). Something gone awry: unsolved mysteries in the evolution of asymmetric animal genitalia. Anim. Biol..

[5] Hedrick BP, Antalek-Schrag P, Conith AJ, Natanson LJ, Brennan PLR (2019). Variability and asymmetry in the shape of the spiny dogfish vagina revealed by 2D and 3D geometric morphometrics. J. Zool..

[6] Romano D (2016). Lateralized courtship in a parasitic wasp. Laterality.

[7] Keener W, Webber MA, Szczepaniak ID, Markowitz TM, Orbach DN (2018). The sex life of harbor porpoises (*Phocoena phocoena*): lateralized and aerial behavior. Aquat. Mamm..

[8] Aronson LR, Clark E (1952). Evidences of ambidexterity and laterality in the sexual behavior of certain poeciliid fishes. Am. Nat..

[9] Neville, A. C. Animal asymmetry. *The Institute of Biology’s Studies on Biology*. *N*. *67*. (Edward Arnold, 1976).

[10] Neville AC (1978). On the general problem of asymmetry. Behav. Brain Sci..

[11] Petersen AD, Lombardo MP, Power HW (2001). Left-sided directional bias of cloacal contacts during tree swallow copulations. Anim. Behav..

[12] Kamimura, Y., Yang, C. C. S. & Lee, C. Y. Fitness advantages of the biased use of paired laterally symmetrical penises in an insect. *J*. *Evol*. *Biol*. (2019). 10.1111/jeb.13486 31081978

[13] Brennan PLR (2007). Coevolution of male and female genital morphology in waterfowl. PLoS One.

[14] Brennan PLR, Clark CJ, Prum RO (2010). Explosive eversion and functional morphology of the duck penis supports sexual conflict in waterfowl genitalia. Proc. Roy. Soc. Lond. B..

[15] Brennan PLR, Prum RO (2015). Mechanisms and evidence of genital coevolution: the roles of natural selection, mate choice and sexual conflict. Cold Spring Harbor Persp. Biol..

[16] Orbach DN, Marshall CD, Mesnick SL, Würsig B (2017). Patterns of cetacean vaginal folds yield insights into functionality. PloS One.

[17] Orbach DN, Hedrick B, Würsig B, Mesnick SL, Brennan PLR (2018). The evolution of genital shape variation in female cetaceans. Evolution.

[18] Slijper, E. J. “Functional morphology of the reproductive system in cetacean” In *Whales*, *Dolphins*, *and Porpoises*, N. S. Norris, Ed. (University of California Press, 1966).

[19] Meek A (1918). The reproductive organs of cetacea. J. Anat..

[20] Orbach, D. N. “Reproductive anatomy” in *Encyclopedia of Marine Mammals*, B. Würsig, J. G. M. Thewissen, K. M. Kovacs, Eds. (Academic Press, 2018).

[21] Orbach DN, Kelly DA, Solano M, Brennan PLR (2017). Genital interactions during simulated copulation among marine mammals. Proc. R. Soc. B.

[22] Orbach DN, Rattan S, Hogan M, Crosby AJ, Brennan PLR (2019). Biomechanical properties of female dolphin reproductive tissue. Acta Biomater..

[23] MacNeilage PF (2014). Evolution of the strongest vertebrate rightward action asymmetries: marine mammal sidedness and human handedness. Psychol. Bull..

[24] Huber BA, Sinclair BJ, Schmitt M (2007). The evolution of asymmetric genitalia in spiders and insects. Biol. Rev..

[25] Orbach DN, Marshall CD, Würsig B, Mesnick SL (2016). Variation in female reproductive tract morphology of the common bottlenose dolphin (*Tursiops truncatus*). Anat. Rec..

[26] Rohlf, F. J. tpsDig, digitize landmarks and outlines, version 2.05. Department of Ecology and Evolution, State University of New York, Stony Brook, New York (2006).

[27] Carpenter, K. E. “Morphometric pattern and feeding mode in emperor fishes (*Lethrinidae*, *Perciformes*)” in *Advances in Morphometrics*, L. F. Marcus, M. Corti, A. Loy, G. J. P. Naylor, D. E. Slice, Eds. (Spriner, 1996).

[28] Arnqvist G, Mårtensson T (1998). Measurement error in geometric morphometrics: empirical strategies to assess and reduce its impact on measures of shape. Acta Zool. Acad. Sci. H..

[29] R Core Team, R: A language and environment for statistical computing. R Foundation for Statistical Computing, Vienna, Austria. URL, https://www.R-project.org/ accessed November (2018).

[30] Zelditch, M. L., Swiderski, D. L., Sheets, H. D., Fink, W. L. *Geometric Morphometrics for Biologists:* A Primer. 2 edn. (Elsevier, 2012).

[31] Adams DC, Otárola-Castillo E (2013). Geomorph: an R package for the collection and analysis of geometric morphometric shape data. Methods Ecol. Evol..

[32] Perez SI, Bernal V, Gonzalez PN (2006). Differences between sliding semi-landmark methods in geometric morphometrics, with an application to human craniofacial and dental variation. J. Anat..

[33] Mitteroecker P, Gunz P, Bernhard M, Schaefer K, Bookstein FL (2004). Comparison of cranial ontogenetic trajectories among great apes and humans. J. Hum. Evol..

[34] Drake AG, Klingenberg CP (2008). The pace of morphological change: historical transformation of skull shape in St Bernard dogs. Proc. Roy. Soc. Lond. B..

[35] Klingenberg CP, McIntyre GS (1998). Geometric morphometrics of developmental instability: analyzing patterns of fluctuating asymmetry with Procrustes methods. Evolution.

[36] Mardia KV, Bookstein FL, Moreton IJ (2000). Statistical assessment of bilateral symmetry of shapes. Biometrika.

[37] M. Ahmed M (1978). Development of asymmetry in the fiddler crab *Uca cumulanta Crane*, 1943 (Decapoda Brachyura). Crustaceana.

[38] Olejnik S, Algina J (2003). Generalized eta and omega squared statistics: measures of effect size for some common research designs. Psychol. Methods.

[39] Brennan PLR (2016). Studying genital coevolution to understand intromittent organ morphology. Integrat. Compar. Biol..

[40] Schneider CA, Rasband WS, Eliceiri KW (2012). NIH Image to ImageJ: 25 years of image analysis. Nat. Methods.

[41] Crossman CA, Taylor EB, Barrett-Lennard LG (2016). Hybridization in the Cetacea: Widespread occurrence and associated morphological, behavioral, and ecological factors. Ecol. Evol..

[42] Orbach DN, Keener W, Ziltener A, Packard J, Würsig B (2019). Testes size, vaginalcomplexity, and behavior in toothed whales (odontocetes): Arms race or tradeoff model for dusky dolphins (*Lagenorhynchus obscurus*), harbor porpoises (*Phocoena phocoena*), and bottlenose dolphins (*Tursiops spp*.*)?*. J. Compar. Psych..

[43] Arnqvist G, Rowe L (2002). Correlated evolution of male and female morphologies in water striders. Evolution; Int. J. Organic Evol..

[44] Fontaine PM, Barrette C (1997). Megatestes: anatomical evidence for sperm competition in the harbor porpoise. Mammalia.

[45] Dines JP (2014). Sexual selection targets cetacean pelvic bones. Evolution.

[46] Eberhard, W. G. *Sexual Selection and Animal Genitalia* (Harvard University Press, 1985).

[47] Ah-King M, Barron AB, Herberstein ME (2014). Genital evolution: why are females still understudied. PLoS One.

[48] Macagno ALM (2011). Shape - but not size - codivergence between male and female copulatory structures in Onthophagus beetles. PLoS One.

[49] Showalter I, Todd BD, Brennan PLR (2014). Intraspecific and interspecific variation of female genitalia in two species of watersnake. Biol. J. Linn. Soc..

[50] Seligmann H (1998). Evidence that minor directional asymmetry is functional in lizard hindlimbs. J. Zool..

[51] Leamy LJ, Klingenberg CP (2005). The genetics and evolution of fluctuating asymmetry. Annu. Rev. Ecol. Evol. Syst..

[52] Dongen SV (2006). Fluctuating asymmetry and developmental instability in evolutionary biology: past, present and future. J. Evol. Biol..

[53] Zelditch ML, Wood AR, Bonett RM, Swiderski DL (2008). Modularity of the rodent mandible: integrating bones, muscles, and teeth. Evol. Dev..

[54] Galatius A, Jespersen A (2005). Bilateral directional asymmetry of the appendicular skeleton of the harbor porpoise (*Phocoena phocoena*). Mar. Mamm. Sci..

[55] Huggenberger S, Rauschmann MA, Vogl TJ, Oelschlager HHA (2009). Functional morphology of the nasal complex in the harbor porpoise (*Phocoena phocoena* L.). Anat. Rec..

[56] Madsen PT, Wisniewska D, Beedholm K (2010). Single source sound production and dynamic beam formation in echolocating harbour porpoises (*Phocoena phocoena)*. J. Exp. Biol..

